# A Narrative Review of Local Therapies in Diabetic Foot Ulcers: Promises, Limitations and Unmet Needs

**DOI:** 10.1155/jdr/9428774

**Published:** 2026-09-23

**Authors:** Dured Dardari

**Affiliations:** ^1^ Diabetology Department, Centre Hospitalier Sud Francilien, Corbeil-Essonnes, France; ^2^ Laboratoire de Biologie de l′Exercice pour la Performance et la Santé, Université d′Évry, IRBA, Université Paris-Saclay, Évry, France, universite-paris-saclay.fr; ^3^ Kremlin-Bicêtre Medical School, Université Paris-Saclay, Le Kremlin-Bicêtre, France, universite-paris-saclay.fr

**Keywords:** diabetic foot ulcer, fish skin, local therapy, negative pressure wound therapy, oxygen therapy, placenta, wound healing

## Abstract

**Background:**

Diabetic foot ulcers (DFUs) frequently remain open despite multidisciplinary standard care. Local therapies target different components of the wound microenvironment, but their comparative value and generalisability are uncertain.

**Objective:**

To critically summarise evidence for seven local approaches—autologous leucocyte–platelet–fibrin patches, placenta‐derived products, sucrose octasulfate dressings, fish skin matrices, hyperbaric oxygen therapy (HBOT), topical oxygen therapy (TOT) and negative pressure wound therapy (NPWT)—and to identify limitations and research priorities.

**Methods:**

PubMed and the Cochrane Library were searched through 31 July 2026, supplemented by citation tracking. Randomised and prospective comparative studies, systematic reviews or meta‐analyses, guidelines and economic analyses were eligible. The narrative synthesis followed SANRA‐informed principles. Forty‐one publications were included, among them 18 randomised trial reports.

**Results:**

An international trial of the leucocyte–platelet–fibrin patch reported healing at 20 weeks in 34% versus 22% with standard care. A meta‐analysis of 11 placental‐product trials reported complete healing in 66.9% versus 34.1% (risk ratio 2.00). EXPLORER reported 48% versus 30% closure with sucrose octasulfate at 20 weeks. In deep ulcers, ODIN reported 44% versus 26% healing with intact fish skin at 16 weeks. TOT meta‐analysis suggested benefit (risk ratio 1.59), whereas HBOT trials were discordant and NPWT evidence was clinically supportive but low certainty. Severe infection, osteomyelitis and critical ischaemia were commonly excluded.

**Conclusions:**

Several local therapies improve healing in selected DFUs, but none replaces debridement, offloading, infection control and vascular care. Product‐specific selection, durable outcomes, head‐to‐head pragmatic trials, complex‐ulcer populations and independent economic evaluation are priority needs.

Core Tip

Local therapies have clinically relevant but phenotype‐specific evidence. This review quantifies the principal trial signals, incorporates conflicting findings and published corrections and emphasises that advanced products are adjuncts to verified multidisciplinary standard care rather than stand‐alone solutions.

## 1. Introduction

Diabetes‐related foot ulceration is a common, recurrent and potentially limb‐threatening complication of diabetes. The International Diabetes Federation estimated that 589 million adults were living with diabetes worldwide in 2024, a number projected to increase substantially by 2050 [1]. The cumulative lifetime risk of a foot ulcer may approach one‐third in people with diabetes, and recurrence remains frequent after healing: Approximately 40% of ulcers recur within 1 year and about 65% within 5 years [2, 3]. These events are associated with infection, hospitalisation, lower extremity amputation, premature mortality, impaired quality of life and major expenditure for patients and health systems [2–4].

Diabetic foot ulcers (DFUs) result from interacting biological and mechanical abnormalities. Peripheral neuropathy reduces protective sensation and alters foot biomechanics; peripheral artery disease limits tissue perfusion and oxygen delivery; repetitive pressure perpetuates trauma; and infection can rapidly extend into deep soft tissue or bone. Healing therefore depends on a coordinated standard‐of‐care pathway that includes pressure offloading, sharp or surgical debridement when indicated, infection diagnosis and treatment, vascular assessment and revascularisation, moisture‐balanced wound care, optimisation of glycaemia and comorbidities and regular multidisciplinary review [5–8]. No local product can compensate for failure to correct these determinants.

Nevertheless, many ulcers remain open despite appropriate standard care. Local therapies have consequently been developed to modify the wound microenvironment, provide a biological scaffold, reduce protease activity, improve oxygen availability, manage exudate or stimulate granulation and epithelialisation. The evidence base has expanded, but products and trial populations are heterogeneous, head‐to‐head comparisons are scarce and patients with active infection, osteomyelitis, severe ischaemia or extensive tissue loss are often excluded. These limitations complicate translation to the complex ulcers encountered in daily practice.

This narrative review critically evaluates seven frequently discussed local approaches: autologous leucocyte–platelet–fibrin patches, placenta‐derived products, sucrose octasulfate dressings, fish skin matrices, hyperbaric oxygen therapy (HBOT), topical oxygen therapy (TOT) and negative pressure wound therapy (NPWT). The objectives are to summarise the included clinical evidence, identify areas of concordance and disagreement, distinguish statistically positive findings from clinically generalisable benefit and define priorities for research and implementation.

## 2. Methods

The review was structured in accordance with principles of transparent narrative synthesis and was informed by the Scale for the Assessment of Narrative Review Articles (SANRA) [9]. A focused search of PubMed and the Cochrane Library was updated through 31 July 2026. Backward and forward citation tracking and targeted searches of journal and guideline websites were used to identify linked trial reports, economic analyses, corrections and errata. Search concepts combined ‘diabetic foot’ or ‘diabetic foot ulcer’ with terms for each intervention, including ‘leucocyte fibrin patch’, ‘platelet fibrin patch’, ‘placental membrane’, ‘amnion’, ‘sucrose octasulfate’, ‘fish skin’, ‘hyperbaric oxygen’, ‘topical oxygen’ and ‘negative pressure wound therapy’.

Eligible publications were English‐ or French‐language randomised or prospective comparative studies enrolling adults with DFUs, systematic reviews or meta‐analyses, evidence‐based guidelines and economic or observational analyses that materially informed efficacy, safety, applicability or cost. Mechanistic reviews were included only when needed to explain a therapy. Studies confined to non‐diabetic wounds, conference abstracts without a full report and reports without clinically interpretable wound outcomes were excluded. Because the purpose was critical narrative synthesis rather than effect estimation, no PRISMA flow diagram, duplicate screening procedure, formal risk‐of‐bias instrument or de novo meta‐analysis was undertaken.

The core evidence set comprised 41 publications: 18 randomised trial reports, one prospective multicentre cohort, one observational cohort, seven systematic reviews or meta‐analyses, two economic analyses, four clinical or health‐economic reviews, six guideline/methodology sources and two correction or erratum notices. For each therapy, data were extracted on study design, country and setting, participant phenotype, sample size, comparator, healing definition, follow‐up, safety and economic outcomes. The principal endpoint across studies was complete epithelial closure, generally assessed between 12 and 20 weeks; other outcomes included time to closure, percentage area reduction, amputation, recurrence, adverse events, resource use and cost.

The evidence was dominated by specialist wound or diabetic foot clinics in Europe and North America. Most participants had chronic neuropathic or neuroischaemic ulcers that were clinically uninfected at randomisation and had adequate perfusion for healing. Severe infection requiring urgent surgery, extensive necrosis, uncontrolled osteomyelitis and critical limb ischaemia were commonly excluded. This selection pattern was considered explicitly when interpreting external validity.

Reference status was checked against PubMed indexing and targeted searches of the Retraction Watch database. No cited item was identified as retracted. A published correction to the EXPLORER trial [10] and an erratum to the ODIN trial [11] were reviewed and cited; neither changed the direction of the primary treatment effect used in this review.

## 3. Local Therapies

### 3.1. Autologous Leucocyte–Platelet–Fibrin Patches

Evidence reviewed: one prospective multicentre cohort and one international observer‐masked randomised controlled trial, together representing more than 300 treated participants [12, 13]. The autologous leucocyte–platelet–fibrin patch is produced from a sample of the patient′s venous blood by centrifugation without exogenous activators. The resulting three‐layer patch supplies a fibrin scaffold, platelets and leucocytes that may support cell migration and release mediators involved in inflammation resolution, angiogenesis and tissue repair.

The pivotal trial enrolled 269 people with hard‐to‐heal DFUs in 32 specialist clinics in the United Kingdom, Denmark and Sweden [12]. After randomisation, 132 participants received the patch in addition to standard care and 137 received standard care alone. Complete epithelialisation within 20 weeks occurred in 45 of 132 ulcers (34%) in the patch group and 29 of 134 evaluable ulcers (22%) in the control group; the reported odds ratio was 1.58 (96% confidence interval [CI], 1.04–2.40). Time to healing was also shorter. A preceding Scandinavian multicentre cohort supported feasibility and provided an early clinical signal in ulcers that had failed to progress with conventional care [13].

The principal strength of this evidence is the focus on genuinely hard‐to‐heal ulcers within specialist multidisciplinary pathways. Important limitations are the absence of a blinded patient or treating clinician, the need for repeated venepuncture and dedicated preparation equipment, limited access outside selected systems and no definitive cost‐effectiveness analysis. The pivotal trial was industry funded, which does not invalidate the findings but reinforces the need for independent replication. The patch may be considered for a clean, adequately perfused ulcer that fails to reduce in area despite verified offloading and wound‐bed preparation; evidence is insufficient for uncontrolled infection or untreated ischaemia.

### 3.2. Placenta‐Derived Products

Evidence reviewed: one systematic review and meta‐analysis of 11 randomised trials involving 655 participants, supplemented by four representative multicentre comparative trials illustrating variation in processing methods and products [14–18]. Placenta‐derived products include cryopreserved or dehydrated amnion, chorion and composite membranes. Depending on processing, they may retain extracellular matrix proteins, growth factors, cytokines, viable cells or structural components. These products should not be interpreted as a single interchangeable class.

In the meta‐analysis, complete healing at 12–16 weeks occurred in 66.9% of participants receiving a human amniotic membrane product and 34.1% receiving standard care, corresponding to a pooled risk ratio of 2.00 (95% CI, 1.67–2.39) [14]. Individual trials of cryopreserved placental membrane, dehydrated amnion/chorion membrane and other allografts also reported faster or more frequent closure than standard dressings [15–18]. Some studies reported striking differences, but sample sizes were modest, run‐in periods and offloading protocols varied and product‐specific effects cannot automatically be generalised to all placental preparations.

The evidence most directly applies to noninfected, nonischaemic neuropathic ulcers treated in specialised outpatient wound centres. Trials generally excluded exposed bone, active osteomyelitis, severe peripheral artery disease and uncontrolled infection. Several were sponsored by manufacturers or included investigators with industry relationships. Practical barriers include acquisition cost, storage and handling requirements, reimbursement variability, donor‐screening and tissue‐processing regulations and uncertainty about the number of applications required. Future trials should compare named products under a common standard‐care protocol and include recurrence, patient‐reported outcomes and full economic evaluation.

### 3.3. Sucrose Octasulfate Dressings

Evidence reviewed: one international double‐blind randomised trial with 240 participants and two health‐economic analyses [10, 19–21]. Sucrose octasulfate is incorporated into a lipidocolloid dressing and is intended to reduce excessive matrix metalloproteinase activity, thereby protecting extracellular matrix components and growth factors from degradation. This mechanism is particularly relevant to stalled chronic wounds characterised by persistent protease activity.

The EXPLORER trial recruited adults with noninfected neuroischaemic DFUs from 43 hospitals and specialist clinics in France, Spain, Italy, Germany and the United Kingdom [19]. At 20 weeks, complete closure was achieved in 48% of participants receiving the sucrose octasulfate dressing and 30% receiving the control dressing; the adjusted odds ratio was 2.60 (95% CI, 1.43–4.73). A subsequent correction notice addressed reporting details and is cited separately [10]; it did not reverse the trial′s primary efficacy conclusion. The population was clinically important because peripheral artery disease was present, but people with active infection or more severe tissue destruction were excluded.

Economic analyses based on trial data or decision models suggested that earlier closure may offset part of the dressing cost [20, 21]. Their conclusions depend on local prices, frequency of dressing changes, health‐system perspective and assumptions about resource use. Sucrose octasulfate has the advantage of being integrated into familiar dressing practice without specialised equipment. Its best‐supported use is a noninfected neuroischaemic ulcer receiving appropriate vascular assessment and offloading; evidence should not be extrapolated to untreated critical ischaemia, deep uncontrolled infection or rapidly progressive necrosis.

### 3.4. Fish Skin Matrices

Evidence reviewed: two randomised clinical programmes reported in three primary publications, a meta‐analysis of eight studies and a focused review [11, 22–26]. Acellular fish skin matrices preserve a porous collagen architecture and, in some products, omega‐3‐rich lipids. Proposed effects include provision of a three‐dimensional scaffold, migration of fibroblasts and keratinocytes, modulation of inflammation, angiogenesis and gradual remodelling into host tissue. Industrially processed cod skin has the strongest standardised clinical evidence; experience with other fish species should not be assumed equivalent.

A multicentre blinded randomised trial in 49 participants with chronic, nonresponsive DFUs reported complete closure at 12 weeks in 67% of fish skin–treated ulcers and 32% of controls [22]. The final efficacy and cost analysis of the same trial programme confirmed a higher proportion of healed wounds and presented an economic model suggesting lower annualised costs, although economic findings remain sensitive to product price and treatment assumptions [23]. A 2024 meta‐analysis of eight studies involving 390 participants found a higher likelihood of complete healing with fish skin than with standard care, but included studies were small and clinically heterogeneous [24].

The ODIN trial extended the evidence to 255 people with deep DFUs penetrating to tendon, joint capsule or bone [25]. Participants were randomised to intact fish skin graft or standard care within multidisciplinary pathways. Complete healing at 16 weeks occurred in 44% and 26%, respectively; corresponding rates were 46% versus 32% at 20 weeks and 55% versus 38% at 24 weeks. The hazard ratio for healing was 1.59. An erratum was subsequently published [11]; review of the notice did not change the primary closure rates or the interpretation used here.

Fish skin is therefore notable for evidence in deeper ulcers than those enrolled in many dressing trials. Even so, trial protocols required infection control and adequate management of ischaemia, and the results do not support placement over uncontrolled purulence, untreated necrosis or inadequately debrided bone infection. Other uncertainties include application frequency, comparative effectiveness versus placental matrices or NPWT, product cost, reimbursement and performance in low‐resource settings. The involvement of the present author in the ODIN/KEREFISH trial is disclosed in the competing interests statement.

### 3.5. HBOT

Evidence reviewed: three randomised controlled trials, one large observational cohort and one Cochrane review [27–31]. HBOT exposes the patient to 100% oxygen at pressures greater than atmospheric pressure in a specialised chamber. It increases dissolved plasma oxygen and may enhance oxygen diffusion, leukocyte function, angiogenic signalling and neovascularisation. Treatment is resource intensive and commonly requires 30–40 sessions, making patient selection essential.

Results are inconsistent. The HODFU trial reported improved complete healing at 1 year in selected patients receiving adjunctive HBOT [27]. In contrast, a double‐blind sham‐controlled trial of 107 randomised participants found no significant difference in healing or in meeting predefined amputation criteria at 12 weeks [28]. The DAMO2CLES multicentre trial likewise did not demonstrate a significant overall improvement in limb salvage or complete wound healing when HBOT was added to standard care for ischaemic ulcers, although treatment completion and selected subgroups remain debated [29]. Observational analyses have also failed to show consistent effectiveness and are vulnerable to confounding by indication [30].

The Cochrane review concluded that HBOT might increase short‐term healing in DFUs but found insufficient reliable evidence for sustained healing or major‐amputation reduction [31]. Differences in ulcer severity, perfusion, chamber protocol, sham exposure, treatment adherence and outcome timing probably contribute to discordant findings. HBOT should not be presented as a routine dressing substitute. It may be considered only after vascular evaluation and correction of remediable ischaemia, infection control, debridement and verified offloading, within centres able to assess treatment burden, contraindications and likelihood of completion.

### 3.6. TOT

Evidence reviewed: three randomised controlled trials and two systematic reviews or meta‐analyses [32–36]. TOT delivers oxygen directly to the wound through continuous low‐flow diffusion or intermittent pressurised systems. Unlike HBOT, it does not create systemic hyperoxia and can be used in outpatient or home settings. Proposed effects include support of collagen synthesis, oxidative bacterial killing, angiogenic signalling and epithelial cell activity, although oxygen penetration depends on the device, wound characteristics and local perfusion.

Randomised studies have generally reported higher closure rates than sham or standard care, but effect sizes vary. A double‐blind multicentre study of continuously diffused oxygen reported a higher probability and faster time to closure [32]. In the TWO2 study, 41.7% of ulcers receiving cyclical pressurised topical oxygen healed at 12 weeks compared with 13.5% receiving sham therapy [33]. A subsequent multicentre trial also found a greater proportion of healed ulcers with adjunctive topical oxygen, although adherence, device type and analysis population influenced results [34].

A systematic review found a favourable healing signal but emphasised small studies and heterogeneity [35]. A later meta‐analysis reported a pooled risk ratio for healing of 1.59 (95% CI, 1.07–2.37), with most participants having Wagner Grade 1–2, noninfected, nonischaemic ulcers [36]. Consequently, the pooled result should not be extrapolated to severe ischaemia or uncontrolled infection. TOT is attractive because it is portable and avoids chamber attendance, but device‐specific evidence, patient adherence, consumable cost and comparative effectiveness remain unresolved.

### 3.7. NPWT

Evidence reviewed: three major randomised trial reports and a Cochrane review encompassing 11 trials and 972 participants [37–40]. NPWT applies controlled subatmospheric pressure through a sealed wound interface. It removes exudate, reduces oedema, promotes macro‐ and microdeformation, supports perfusion at the wound edge and stimulates granulation tissue. Its clinical role is distinct from that of a passive dressing and is particularly relevant after surgical debridement or partial foot amputation.

In a multicentre trial of postamputation wounds, NPWT increased the proportion of wounds reaching complete closure and accelerated granulation compared with standard moist wound care [37]. A second multicentre trial in chronic DFUs also reported more complete closure and fewer secondary amputations with vacuum‐assisted closure than with advanced moist wound therapy [38]. However, the pragmatic German DiaFu trial did not find a statistically significant difference in complete wound closure in the intention‐to‐treat analysis, although per‐protocol analyses suggested shorter healing among participants who completed assigned treatment [40].

The Cochrane review judged that NPWT may improve healing after amputation and in DFUs but rated the certainty of evidence as low because of risk of bias, imprecision and heterogeneous protocols [39]. NPWT requires trained staff, an adequate seal, regular reassessment and careful protection of vessels, nerves and exposed structures. It is contraindicated or inappropriate in several settings, including untreated necrotic tissue, inadequately controlled osteomyelitis and unprotected exposed vessels. It should be used as one phase of a wound strategy—for example, to prepare a debrided wound for delayed closure or grafting—rather than continued automatically without objective progress.

The comparative evidence, participant profiles, clinical signals and principal limitations are summarised in Table 1.

**Table 1:** Comparative evidence for innovative local therapies in diabetic foot ulcers.

Therapy	Evidence reviewed	Typical participants and settings	Main clinical signal	Principal limitations	Key references
Leucocyte–platelet–fibrin patch	one prospective cohort; one international RCT (> 300 participants)	Hard‐to‐heal, adequately perfused DFUs in specialist UK, Danish and Swedish clinics	34% vs. 22% healed at 20 weeks; shorter time to healing	Venepuncture and preparation device; limited availability; no definitive cost‐effectiveness; industry funding	[12, 13]
Placenta‐derived products	Meta‐analysis of 11 RCTs (655 participants) plus four representative comparative trials	Mostly noninfected, nonischaemic neuropathic DFUs in US wound centres	Pooled complete healing 66.9% vs. 34.1%; RR 2.00	Marked product heterogeneity; small trials; frequent industry involvement; uncertain class effect and cost	[14–18]
Sucrose octasulfate dressing	one international RCT (*n* = 240) plus two economic analyses	Noninfected neuroischaemic DFUs in 43 European specialist centres	48% vs. 30% closed at 20 weeks; adjusted OR 2.60	Not tested in active infection or untreated critical ischaemia; model‐dependent economic results	[10, 19–21]
Fish skin matrix	two randomised programmes (three reports); meta‐analysis of eight studies (*n* = 390)	Chronic nonresponsive DFUs and deep ulcers reaching tendon, capsule or bone	67% vs. 32% at 12 weeks in a small RCT; 44% vs. 26% at 16 weeks in ODIN	Product‐specific evidence; cost/reimbursement; infection must be controlled; limited head‐to‐head data	[11, 22–26]
Hyperbaric oxygen therapy	three RCTs; one observational cohort; one Cochrane review	Selected chronic or ischaemic DFUs treated in specialised chambers	Positive 1‐year healing signal in one trial; other sham‐controlled/pragmatic trials neutral	High resource and patient burden; heterogeneous protocols; uncertain durable healing and amputation benefit	[27–31]
Topical oxygen therapy	three RCTs; two systematic reviews/meta‐analyses	Mainly Wagner Grade 1–2, noninfected, nonischaemic outpatient DFUs	Pooled healing RR 1.59; positive device‐specific trials	Different devices/doses; adherence and blinding issues; limited severe‐ulcer evidence	[32–36]
Negative pressure wound therapy	three major RCT reports; Cochrane review of 11 trials (*n* = 972)	Postdebridement, postamputation or chronic DFUs requiring exudate control and granulation	Several trials favoured closure or time to healing; DiaFu ITT analysis neutral	Low‐certainty evidence; protocol heterogeneity; equipment and expertise; contraindications in untreated necrosis/osteomyelitis	[37–40]

Abbreviations: CI, confidence interval; DFU, diabetic foot ulcer; ITT, intention‐to‐treat; OR, odds ratio; RCT, randomised controlled trial; RR, risk ratio.

**Figure 1:**
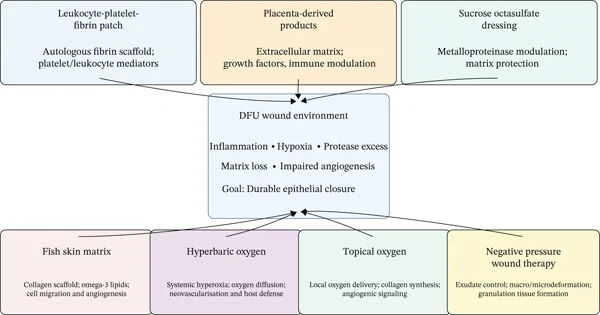
Mechanistic pathways targeted by innovative local therapies in diabetic foot ulcers. The figure was created by the author as a conceptual synthesis; it does not imply equivalent efficacy across therapies.

The principal mechanistic pathways targeted by these therapies are shown in Figure 1. The diagram is a conceptual synthesis based on the clinical and mechanistic literature cited in this review [8, 12, 14, 19, 22, 27, 32, 37].

## 4. Discussion

This review identifies credible healing signals for several local therapies, but no intervention can be designated a universal best treatment. The strongest conclusions are product‐ and phenotype‐specific. The leucocyte–platelet–fibrin patch improved healing in hard‐to‐heal ulcers managed in specialist clinics [12]; placenta‐derived products increased closure in selected noninfected, adequately perfused ulcers [14–18]; sucrose octasulfate improved healing in noninfected neuroischaemic ulcers [19]; intact fish skin improved closure in both chronic superficial ulcers and deeper ulcers with exposed structures after appropriate wound preparation [11, 22–25]; TOT showed a pooled benefit in mainly mild‐to‐moderate ulcers [32–36]; and NPWT retained an important role after debridement or partial amputation despite low‐certainty comparative evidence [37–40]. HBOT remains the most visibly discordant evidence base, with positive and neutral trials [27–31].

A 2026 systematic review and network meta‐analysis included 51 randomised trials, 6161 participants and 23 topical interventions [41]. Such analyses are valuable for mapping a fragmented field, but rankings are driven largely by indirect comparisons and inherent variation in ulcer phenotype, follow‐up, outcome definitions, standard care and risk of bias. They should therefore generate hypotheses rather than justify interchangeable use of distinct products. Direct pragmatic comparisons between leading therapies, layered on a rigorously standardised care pathway, remain a major unmet need.

The most clinically useful framework is not simply ‘advanced therapy versus standard care’ but selection according to wound phenotype and treatment phase. A clean neuropathic ulcer that has failed to progress after verified offloading may be a candidate for a biological patch or matrix. A noninfected neuroischaemic ulcer may fit the EXPLORER population for sucrose octasulfate, provided vascular assessment has been completed. NPWT may be most appropriate after extensive debridement or partial amputation, whereas portable TOT may be considered when adherence to outpatient therapy is feasible. HBOT requires a separate decision that weighs tissue oxygenation, revascularisation options, comorbidity, treatment burden and access to a chamber. This framework is interpretive, not a substitute for guidelines or individual multidisciplinary assessment [5–8].

External validity is the central weakness across the literature. Trials commonly exclude active infection needing surgery, uncontrolled osteomyelitis, extensive gangrene, severe renal or cardiac disease and critical limb‐threatening ischaemia. Yet these features frequently coexist in referral practice. The ODIN trial partly narrowed this gap by enrolling ulcers extending to tendon, joint capsule or bone [25], but infection still had to be controlled and standard care remained intensive. Trials in complex postdebridement wounds, infected ulcers after source control and resource‐limited settings are therefore priorities.

Heterogeneity in standard care also threatens interpretation. Offloading is among the most powerful determinants of healing, but device choice, enforcement, adherence monitoring, debridement frequency, vascular thresholds and run‐in criteria vary substantially [6]. An apparently effective local product may compensate for suboptimal control care, whereas an effective product may appear neutral when used late or inconsistently. Future protocols should prespecify offloading, infection treatment, revascularisation, debridement, dressing frequency, rescue criteria and cointerventions, and should report adherence in both groups.

Outcome selection should move beyond closure at a single time point. Time to durable closure, recurrence, infection‐related hospitalisation, minor and major amputation, pain, mobility, quality of life, treatment burden, adverse events and days at home are meaningful to patients and health systems. Blinded confirmation of closure and a period of sustained closure should be routine. Because many DFUs recur, a product that accelerates epithelialisation without improving durability may have less value than suggested by a 12‐week endpoint [3].

Economic evidence is incomplete and frequently model‐based. Acquisition price alone is misleading: A costly product may be efficient if it shortens nursing time, avoids hospitalisation or prevents surgery, whereas a relatively inexpensive dressing may be wasteful if prolonged without progress. Analyses should use transparent local prices, include offloading and staff time, report the number of applications, distinguish payer and societal perspectives and perform sensitivity analyses. Independent health‐technology assessments are particularly important for biologics, devices and chamber‐based therapy.

Industry involvement is common in advanced wound‐care trials because product development and multicentre research are expensive. The appropriate response is not to dismiss industry‐funded evidence but to require prospective registration, concealed allocation, blinded outcome assessment, complete reporting, independent statistical analysis where possible, access to protocols and replication by groups without financial dependence on the manufacturer. Product‐specific conflicts should be transparently disclosed.

This review has limitations. It is a narrative review rather than a systematic review; study screening and extraction were not performed independently by two reviewers, and no formal risk‐of‐bias assessment or de novo meta‐analysis was undertaken. Restriction to English and French may have excluded relevant reports. The number of studies quoted for each therapy reflects the core evidence selected for clinical interpretation and is not an exhaustive product inventory. Finally, rapid publication in this field means that evidence and regulatory availability may change. These limitations are offset partly by explicit search methods, inclusion of contradictory findings, attention to corrections and errata and integration of guideline, clinical and economic perspectives.

## 5. Conclusion

Local therapies can improve healing in selected DFUs when they are added to high‐quality multidisciplinary care, but benefits are not uniform across products or wound phenotypes. Current evidence is most persuasive for defined populations: hard‐to‐heal clean ulcers for autologous leucocyte–platelet–fibrin patches; selected noninfected ulcers for named placenta‐derived products; noninfected neuroischaemic ulcers for sucrose octasulfate; appropriately prepared chronic or deep ulcers for intact fish skin; selected mild‐to‐moderate ulcers for TOT; and postdebridement or postamputation wounds for NPWT. Evidence for HBOT remains conflicting. Future research should prioritise pragmatic head‐to‐head trials, complex and infected ulcers after source control, durable healing, recurrence, patient‐centred outcomes and independent cost‐effectiveness analyses. The clinical value of any local therapy ultimately depends on correct wound selection, timely use and integration with offloading, debridement, infection control, vascular care and metabolic optimisation.

## Author Contributions

**Dured Dardari:** conceptualisation, methodology, investigation, resources, data curation, writing – original draft, writing – review and editing, visualisation, supervision, project administration.

## Funding

No funding was received for this manuscript.

## Disclosure

The author had read and approved the final version of the manuscript. Dured Dardari, the corresponding author and manuscript guarantor, had full access to all of the data in this study and takes complete responsibility for the integrity of the data and the accuracy of the data analysis. The cited references were checked for retraction status on 7 August 2026 using PubMed and targeted Retraction Watch searches. No cited publication was identified as retracted. The EXPLORER correction [10] and ODIN erratum [11] were reviewed and do not change the principal interpretation presented in this article.

## Ethics Statement

This article is a narrative review of previously published, nonidentifiable data. No new participants, human tissue, personal health information or animals were enrolled or studied; therefore, institutional ethical approval and consent to participate were not required.

## Consent

Not applicable. The manuscript contains no identifiable individual‐level information.

## Conflicts of Interest

The author declares no conflicts of interest.

## Data Availability

The data that support the findings of this study are available from the corresponding author upon reasonable request.
